# Clinical impact of lipid injectable emulsion in internal medicine inpatients exclusively receiving parenteral nutrition: a propensity score matching analysis from a Japanese medical claims database

**DOI:** 10.1186/s12916-022-02568-x

**Published:** 2022-10-27

**Authors:** Kosei Takagi, Kenta Murotani, Satoru Kamoshita, Akiyoshi Kuroda

**Affiliations:** 1 Department of Gastroenterological Surgery, Dentistry, and Pharmaceutical Sciences, Okayama University Graduate School of Medicine, 2-5-1 Shikata-cho, Kita-ku, Okayama, 700-8558 Japan; 2 Biostatistics Center, Kurume University, Kurume, Japan; 3 Medical Affairs Department, Research and Development Center, Otsuka Pharmaceutical Factory, Inc, Tokyo, Japan; 4 Research and Development Center, Otsuka Pharmaceutical Factory, Inc, Tokyo, Japan

**Keywords:** Parenteral nutrition, Lipid injectable emulsion, Medical inpatient, Clinical outcome, Real-world data

## Abstract

**Background:**

Although guidelines recommend lipid injectable emulsions (ILEs) be used as a part of parenteral nutrition, many patients in Japan receive lipid-free parenteral nutrition. Furthermore, little is known about the effect of ILEs on clinical outcomes in medical inpatients managed with parenteral nutrition. The aim of this study was to investigate the clinical impact of ILEs on internal medicine inpatients receiving parenteral nutrition.

**Methods:**

A propensity score matching (PSM) analysis was performed using a medical claims database covering 451 hospitals in Japan. Participants included the following internal medicine inpatients, ages ≥ 18 years, fasting > 10 days, and receiving exclusively parenteral nutrition, between 2011 and 2020. Participants were divided into 2 groups: those who did and did not receive ILEs. The primary endpoint was in-hospital mortality. The secondary endpoints included intravenous catheter infection, activities of daily living (ADL), hospital length of stay (LOS), and total medical costs. To adjust for energy doses, logistic or multiple regression analyses were performed using energy dose as an additional explanatory variable.

**Results:**

After PSM, 19,602 matched pairs were formed out of 61,437 patients. The ILE group had significantly lower incidences than the non-ILE group of in-hospital mortality (20.3% vs. 26.9%; odds ratio [OR], 0.69; 95% confidence interval [CI], 0.66–0.72; *p* < 0.001), deteriorated ADL (10.8% vs. 12.5%; OR, 0.85; 95% CI, 0.79–0.92; *p* < 0.001), and shorter LOS (regression coefficient, − 0.8; 95% CI, − 1.6–0.0; *p* = 0.045). After adjusting for energy dose, these ORs or regression coefficients demonstrated the same tendencies and statistical significance. The mean total medical costs were $21,009 in the ILE group and $21,402 in the non-ILE group (*p* = 0.08), and the adjusted regression coefficient for the ILE vs. the non-ILE group was − $860 (95% CI, − $1252 to − $47).

**Conclusions:**

ILE use was associated with improved clinical outcomes, including lower in-hospital mortality, in internal medicine inpatients receiving parenteral nutrition.

**Supplementary Information:**

The online version contains supplementary material available at 10.1186/s12916-022-02568-x.

## Background

Lipid injectable emulsions (ILEs) serve as a source of essential fatty acids and energy-dense non-protein calories, as well as a principal part of parenteral nutrition [1, 2]. Nutritional support has been associated with improved clinical outcomes in hospitalized patients [3, 4]. Recent American Society for Parenteral and Enteral Nutrition (ASPEN) recommendations have advocated for the use of ILEs for those patients who require parenteral nutrition [5]. This recommendation is based on the potential clinical and biochemical benefits of the addition of ILEs to parenteral nutrition, which include modulation of inflammatory responses and reduction of immune suppression.

The value of using ILEs as part of parenteral nutrition for surgical and critically ill patients has been well established, with specific ILEs demonstrating both therapeutic and adverse effects [6]. On the other hand, recent retrospective surveys using medical claims databases [7, 8] have suggested that ILEs are not being widely used as part of parenteral nutrition in the current clinical practice in Japan, though this is not the case globally. Moreover, little is known about the effects of ILEs on internal medicine inpatients who are being managed with parenteral nutrition. In particular, real-world data on the actual impact of ILEs on clinical outcomes in internal medicine inpatients being managed with parenteral nutrition are lacking, and there have been no studies that have investigated the cost-effectiveness of using ILEs for these patients.

Clarifying the impact of ILEs on clinical outcomes in internal medicine inpatients may help promote the more appropriate management of parenteral nutrition in this patient population. The purpose of this study was to examine the impact of the use of ILEs on clinical outcomes (i.e., mortality, activities of daily living, and complications) and medical costs in adult internal medicine inpatients receiving parenteral nutrition, using a medical claims database.

## Methods

### Design and data source

A retrospective analysis was performed using data that was extracted from a medical claims database which included 451 hospitals and managed by the Medical Data Vision Co., Ltd. (MDV; Tokyo, Japan). The database uses the diagnosis procedure combination/per-diem payment system (DPC/PDPS), in which provider reimbursement is calculated on a flat-rate per-diem fee based on the diagnosis group. The study protocol was approved by the Ethics Committees of the Okayama University Graduate School of Medicine, Dentistry, and Pharmaceutical Sciences (No. 2108-041) and the Kurume University Graduate School of Medicine (No. 21139) and registered at the University Hospital Medical Information Network Clinical Trial Registry (UMIN000044962). Informed consent was not required, because all personal information used in this study was anonymized.

The database included information on dates of hospital admission and discharge, age at admission, sex, height, body weight, body mass index (BMI), number of beds in admission hospital, year and type of admission, primary diseases (coded using the International Statistical Classification of Diseases and Related Health Problems, 10th Revision [ICD-10]), comorbidities (used to determine the Charlson Comorbidity Index [CCI]) [9], activities of daily living (ADL) based on the Barthel Index (BI) [10], levels of consciousness based on the Japan Coma Scale (JCS) [11], malnutrition defined as having poor oral intake of at least 10 days and low body mass index according to the Global Leadership Initiative on Malnutrition (GLIM) criteria [12], medical treatments during hospitalization (using Japan-specific medical claims codes), and discharge outcome status, as well as other information not used in our study. The total daily doses of parenteral energy, amino acids, and ILE prescribed were calculated using the parenteral nutrition infusion product names and compositions along with the prescribed quantities of those products, as they appeared in the database. When recording these doses, day 1 was regarded as the day fasting started, day 2 as the second day after fasting started, and so on.

### Patient population

This study included hospitalized adult patients ages 18 years or older who were fasting (receiving no oral or enteral nutrition) for more than 10 consecutive days and were managed with parenteral nutrition, between January 2011 and September 2020. Patients were excluded from the study who underwent surgery or entered the intensive care unit between the day of admission and the start of fasting, were suspected to be in the terminal disease phase (defined as prescribed mean energy doses < 10 kcal/kg or mean amino acid doses < 0.5 g/kg on days 4 through 10), or were considered to be overfed (which we based on prescribed mean energy doses ≥ 30 kcal/kg on days 4 through 10). The rationale for the use of days 4 through 10 was that the administration of parenteral nutrition usually involves a gradual increase in dose over the initial 3 to 4 days before reaching the full target dose [7, 8].

### Clinical outcomes

The primary endpoint was in-hospital mortality. The secondary endpoints included intravenous catheter infection during hospitalization, deteriorated ADL at discharge, length of stay (LOS), readmission, and total medical costs. ADL at discharge, LOS, and readmission were recorded for only those patients who were discharged alive, whereas other data were recorded for all patients. Medical costs were calculated based on Japanese yen and were then converted to US dollar (US$) using the annual exchange rate of 2020 reported by the Organization for Economic Cooperation and Development (OECD) (US$1 = 107 Japanese yen) [13]. Patients were considered to have deteriorated ADL when their total BI scores were lower at the time of discharge than at the time of admission. Readmission was defined as being admitted to the same hospital again within 30 days of discharge.

### Variables

The variables extracted from the database were categorized as follows: age at admission (18–59, 60–69, 70–79, 80–89, or ≥ 90 years), BMI (< 16.0, 16.0–18.5, 18.5–22.5, 22.5–25.0, or ≥ 25.0), number of beds admission hospital (< 200, 200–500, or ≥ 500), year of admission (2011–2012, 2013–2014, 2015–2016, 2017–2018, or 2019–2020), type of admission (elective or emergency), primary disease (by ICD-10 code), comorbidities (CCI of 0, 1, 2, or ≥ 3), ADL (BI of 0, 5–20, 25–40, 45–60, 65–95, or 100), levels of consciousness (JCS of 0 [alert], 1–3 [awake], 10–30 [arousable], or 100–300 [coma]), and nutritional status (malnutrition defined as BMI < 18.5 if < 70 years old or BMI < 20 if > 70 years old). Information about medical treatments (e.g., albumin infusion, blood transfusion, respirator use, dialysis, nutrition support team, and rehabilitation) ordered between the day of admission and day 10 was extracted from the database for each patient. Missing values for the type of admission, BI, and JCS were placed in an “unknown” category.

### Prescribed doses of parenteral nutrition

The prescribed mean daily doses of energy, amino acids, and ILE for days 4 to 10 after the start of fasting were calculated for each patient based on the parenteral nutrition infusion product composition and prescribed quantity of that infusion and were based on the assumption that nutrient doses often take until day 4 to reach 100% of their target [14]. Prescribed daily doses of energy and amino acids were calculated as kilocalories (kcal) and grams (g), respectively, and reported per kilogram (kg) of body weight and prescribed daily doses of ILE were calculated and reported as both grams and the caloric percentage (%) of the total non-protein energy administered that day.

### Statistical analysis

The data management and statistical analysis were performed by an independent third party (A2 Healthcare Corporation; Tokyo, Japan) in order to eliminate arbitrariness and ensure transparency. Categorical variables were summarized as numbers and percentages, and continuous variables were summarized as means and standard deviations (SD). Missing values were not included. First, patients eligible for the study were divided into 2 groups: the ILE group, who were prescribed ILEs during days 4 through 10, and the non-ILE group, who were not prescribed ILEs during days 4 through 10. Next, propensity score matching (PSM) was used to adjust for confounding factors [15]. The propensity score was estimated by multivariable logistic regression analysis with the ILE group as the objective variable and patient characteristics as the explanatory variables. PSM was conducted using a one-to-one nearest neighbor method and using the caliper width. The caliper value was 0.2, and matching was performed within the caliper values. To confirm the covariate balance between the groups, standardized differences were calculated before and after PSM. A standardized difference less than 10% was considered to represent a balanced covariate [16].

To compare the 2 groups for each outcome, both before and after PSM, the Student *t*-test was used for continuous variables and the chi-square test was used for categorical variables. To adjust for the differences in the prescribed mean daily parenteral energy doses between the 2 groups, even after PSM, multivariable logistic or multiple regression analyses, as appropriate, were performed, with the mean daily energy dose prescribed for days 4 through 10 added as an explanatory variable. In these analyses, odds ratios (ORs) or regression coefficients, as appropriate, along with 95% confidence intervals (CIs), were calculated, both before and after the adjustment for energy.

For in-hospital mortality, survival curves were generated for the 2 groups using the Kaplan-Meier method, and a log-rank test was performed. In addition, the Cox proportional hazard model was used to calculate a hazard ratio (HR), along with a 95% CI, of the ILE group to the non-ILE group, for in-hospital mortality. For these calculations, the patients who were discharged alive were censored on the day of discharge, and the inpatients who survived for 180 days or longer were censored on day 180. All statistical analyses were performed using SAS version 9.4 (SAS Institute Inc., Cary, NC, USA), with a two-sided significance level of 5%.

### Sensitivity analysis

Before modeling, the variance inflation factors (VIFs) of patient characteristics and prescribed mean daily parenteral nutrition doses were calculated to confirm that there was no multicollinearity between variables based on multiple regression analysis or multivariable logistic regression analysis [17].

To confirm the robustness of PSM, confounding factors were adjusted by multivariable logistic regression analysis or multiple regression analysis, and an adjustment analysis consisting of 2 explanatory variable groups (model 1, model 2) was performed. In model 1, the explanatory variables were the 2 groups and the patient characteristics. In model 2, the explanatory variables were those included in model 1 as well as the prescribed mean daily parenteral energy during days 4 to 10. Either ORs or regression coefficients, along with 95% CIs, were calculated for each model.

## Results

### Patient characteristics

**Fig. 1:**
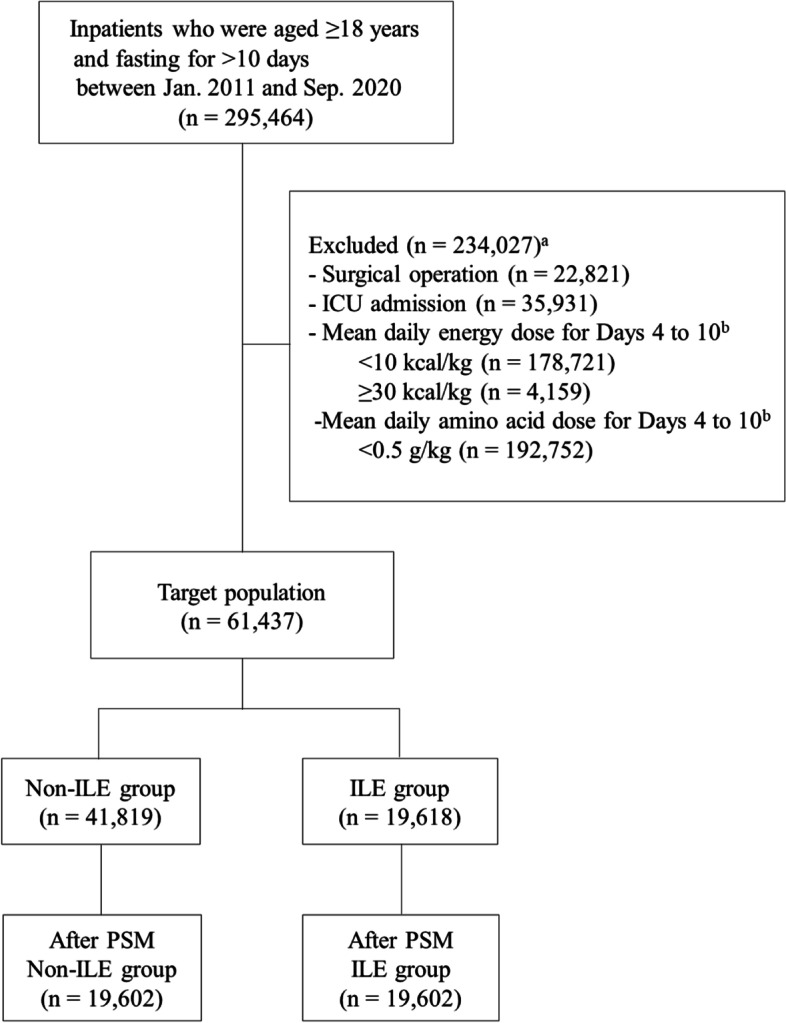
Flowchart of patient disposition in the study. ^a^Some patients had more than one reason for exclusion. ^b^Day 1 is regarded as the day fasting started. *Abbreviations*: ILE, lipid injectable emulsion (soybean oil-based); ICU, intensive care unit; PSM, propensity score matching

Following the screening of 295,464 medical inpatients, a total of 61,437 patients were eligible for the study (Fig. 1). Based on the GLIM criteria, malnutrition was found in 28,097 (45.7%) of the study patients (Table 1). Among all patients, 19,618 (31.9%) were in the ILE group and 41,819 (68.1%) were in the non-ILE group, and the mean (SD) parenteral nutrition duration for all patients was 24.4 (28.5) days. After PSM, 19,602 matched pairs of patients were formed. Of the 19,602 patients in the ILE group, 16,191 (82.6%) were 60 years or older, and 11,439 (58.4%) were males; in addition, the most common primary disease was digestive system malignancy in 6723 (34.3%) patients, followed by digestive system disease in 4865 (24.8%) patients.

**Table 1:** Characteristics of 61,437 internal medicine inpatients aged 18 years or older and fasting for more than 10 days^a^ in Japan, January 2011 to September 2020

Variables	Before PSM			After PSM		
	Non-ILE group (*n* = 41,819)	ILE group (*n* = 19,618)	Standardized difference, %	Non-ILE group (*n* = 19,602)	ILE group (*n* = 19,602)	Standardized difference, %
Age (years)						
18–59	6888 (16.5)	3415 (17.4)	− 4.4	3420 (17.4)	3411 (17.4)	0.1
60–69	8073 (19.3)	3747 (19.1)		3762 (19.2)	3744 (19.1)	
70–79	11,097 (26.5)	5429 (27.7)		5407 (27.6)	5427 (27.7)	
80–89	11,596 (27.7)	5437 (27.7)		5417 (27.6)	5431 (27.7)	
> 90	4165 (10.0)	1590 (8.1)		1596 (8.1)	1589 (8.1)	
Sex						
Male	22,131 (52.9)	11,453 (58.4)	− 11.0	11,475 (58.5)	11,439 (58.4)	0.4
Female	19,688 (47.1)	8165 (41.6)		8127 (41.5)	8163 (41.6)	
Body mass index (kg/m^2^)						
<16	5350 (12.8)	2793 (14.2)	− 6.3	2797 (14.3)	2790 (14.2)	− 0.1
16–18.5	9193 (22.0)	4516 (23.0)		4464 (22.8)	4513 (23.0)	
18.5–22.5	16,613 (39.7)	7673 (39.1)		7680 (39.2)	7668 (39.1)	
22.5–25	6050 (14.5)	2712 (13.8)		2758 (14.1)	2709 (13.8)	
25–30	3847 (9.2)	1650 (8.4)		1642 (8.4)	1648 (8.4)	
≥ 30	766 (1.8)	274 (1.4)		261 (1.3)	274 (1.4)	
Number of hospital beds						
< 200	4156 (9.9)	1880 (9.6)	6.5	1848 (9.4)	1879 (9.6)	− 0.6
200–500	23,247 (55.6)	10,246 (52.2)		10,226 (52.2)	10,236 (52.2)	
≥ 500	14,416 (34.5)	7492 (38.2)		7528 (38.4)	7487 (38.2)	
Year of admission						
2011–2012	3698 (8.8)	1571 (8.0)	5.6	1577 (8.0)	1571 (8.0)	0.3
2013–2014	7603 (18.2)	3305 (16.8)		3309 (16.9)	3304 (16.9)	
2015–2016	10,543 (25.2)	4854 (24.7)		4885 (24.9)	4850 (24.7)	
2017–2019	11,459 (27.4)	5663 (28.9)		5621 (28.7)	5658 (28.9)	
2019–2020	8516 (20.4)	4225 (21.5)		4210 (21.5)	4219 (21.5)	
Type of admission						
Elective	23,287 (55.7)	11,293 (57.6)	− 0.6	11,212 (57.2)	11,284 (57.6)	− 0.2
Emergency	18,505 (44.3)	8315 (42.4)		8379 (42.7)	8308 (42.4)	
NA	27 (0.1)	10 (0.1)		11 (0.1)	10 (0.1)	
Primary disease						
Digestive system malignancy	10,645 (25.5)	6733 (34.3)	− 8.9	6798 (34.7)	6723 (34.3)	0.6
Hematological malignancy	1647 (3.9)	324 (1.7)		302 (1.5)	324 (1.7)	
Other malignancies	3156 (7.5)	998 (5.1)		1001 (5.1)	998 (5.1)	
Sepsis	675 (1.6)	233 (1.2)		231 (1.2)	233 (1.2)	
Coagulopathy disease	337 (0.8)	112 (0.6)		117 (0.6)	111 (0.6)	
Cerebrovascular disease	1600 (3.8)	413 (2.1)		417 (2.1)	413 (2.1)	
Cardiovascular disease	1501 (3.6)	486 (2.5)		450 (2.3)	486 (2.5)	
Respiratory disease	6057 (14.5)	2812 (14.3)		2785 (14.2)	2808 (14.3)	
Digestive system disease	9556 (22.9)	4866 (24.8)		4879 (24.9)	4865 (24.8)	
Kidney and urinary tract disease	1065 (2.5)	439 (2.2)		453 (2.3)	439 (2.2)	
Others	5580 (13.3)	2202 (11.2)		2169 (11.1)	2202 (11.2)	
Charlson Comorbidity Index						
0	16,361 (39.1)	7255 (37.0)	2.2	241 (36.9)	7253 (37.0)	− 0.2
1	1447 (3.5)	619 (3.2)		625 (3.2)	619 (3.2)	
2	14,532 (34.7)	7677 (39.1)		7630 (38.9)	7663 (39.1)	
≥ 3	9479 (22.7)	4067 (20.7)		4106 (20.9)	4067 (20.7)	
Barthel Index						
100	15,111 (36.1)	8636 (44.0)	− 16.0	8696 (44.4)	8625 (44.0)	0.6
65–95	4022 (9.6)	1897 (9.7)		1909 (9.7)	1897 (9.7)	
45–60	2694 (6.4)	1181 (6.0)		1154 (5.9)	1181 (6.0)	
25–40	1459 (3.5)	586 (3.0)		588 (3.0)	584 (3.0)	
5–20	2510 (6.0)	1050 (5.4)		1017 (5.2)	1050 (5.4)	
0	11,104 (26.6)	4230 (21.6)		4204 (21.4)	4228 (21.6)	
NA	4919 (11.8)	2038 (10.4)		2034 (10.4)	2037 (10.4)	
Japan Coma Scale						
0	31,781 (76.0)	15,775 (80.4)	− 11.5	15,780 (80.5)	15,763 (80.4)	0.8
1–3	5414 (12.9)	2266 (11.6)		2308 (11.8)	2263 (11.5)	
10–30	2269 (5.4)	808 (4.1)		778 (4.0)	808 (4.1)	
100–300	1213 (2.9)	399 (2.0)		393 (2.0)	399 (2.0)	
NA	1142 (2.7)	370 (1.9)		343 (1.7)	369 (1.9)	
Nutritional status^b^						
Malnutrition	18,855 (45.1)	9242 (47.1)	4.1	9225 (47.1)	9234 (47.1)	0.1
Medical treatment^c^						
Albumin infusion	3498 (8.4)	2287 (11.7)	11.0	2235 (11.4)	2280 (11.6)	0.8
Blood transfusion	7145 (17.1)	3418 (17.4)	0.9	3435 (17.5)	3412 (17.4)	− 0.3
Respirator use	1605 (3.8)	1007 (5.1)	6.3	990 (5.1)	1002 (5.1)	0.3
Dialysis	331 (0.8)-	200 (1.0)	2.4	197 (1.0)	199 (1.0)	0.1
Nutrition support team^d^	2268 (5.4)	2170 (11.1)	20.6	1921 (9.8)	2157 (11.0)	4.4
Rehabilitation	13,397 (32.0)	7032 (35.8)	8.1	6882 (35.1)	7023 (35.8)	1.5
PN duration, mean (SD), days	25.1 (30.3)	23.0 (24.3)	− 7.6	24.5 (26.7)	23.0 (24.3)	− 5.4

Data presented as numbers of patients (percentages), unless otherwise indicated

*Abbreviations*: *ILE*, lipid injectable emulsion (soybean oil-based); *NA*, not available; *PSM*, propensity score matching; *PN*, parenteral nutrition; *BMI*, body mass index

^a^Day 1 regarded as the day fasting started

^b^Malnutrition defined as phenotype criteria (BMI < 18.5 if < 70 years old or < 20 if > 70 years old) as well as etiologic criteria (fasting for more than 10 days), based on the GLIM criteria for Asian populations [12]

^c^From the day of admission to day 10

^d^Intervention by the nutrition support team

Before PSM, the standardized differences between the 2 groups were greater than 10% for sex, BI, JCS, and medical treatments of albumin infusion and nutrition support team (Table 1). After PSM, there were no variables with standardized differences greater than 10%.

### Mean daily nutrition doses for days 4 to 10

The prescribed mean daily doses of parenteral nutrition for days 4 to 10 were calculated for both groups, both before and after PSM (Table 2). In the ILE group, both before and after PSM, the mean (SD) non-protein calorie ratio of ILEs was 14.3 (11.5) %, and the mean dose of ILEs was 14.2 (10.9) g. After PSM, the mean (SD) energy dose was 16.5 (4.8) kcal/kg in the non-ILE group and 18.8 (5.1) kcal/kg in the ILE group, and this represented a significant difference (*p* < 0.001). Also after PSM, the mean (SD) daily dose of amino acids was 0.73 (0.17) g/kg in both the non-ILE and ILE groups.

**Table 2:** Daily parenteral nutrition doses on days 4 to 10^a^ prescribed to 61,437 internal medicine inpatients aged 18 years or older and fasting for more than 10 days in Japan, January 2011 to September 2020

Nutrition components	Before PSM			After PSM		
	Non-ILE group (*n* = 41,819)	ILE group (*n* = 19,618)	*p*-value^b^	Non-ILE group (*n* = 19,602)	ILE group (*n* = 19,602)	*p*-value^b^
Amino acids, g/kg	0.74 (0.18)	0.73 (0.17)	< 0.001	0.73 (0.17)	0.73 (0.17)	0.43
Energy, kcal/kg	16.6 (5.0)	18.8 (5.1)	< 0.001	16.5 (4.8)	18.8 (5.1)	< 0.001
Lipids, g	…	14.2 (10.9)	…	…	14.2 (10.9)	…
Lipid energy ratio, %	…	14.3 (11.5)	…	…	14.3 (11.5)	…

Data presented as means (standard deviations)

*Abbreviations*: *PSM*, propensity score matching; *ILE*, lipid injectable emulsion (soybean oil-based)

^a^Day 1 regarded as the day fasting started

^b^Based on the Student *t*-test

### Clinical outcomes

The results for primary and secondary endpoints, before and after PSM, as well as the ORs or regression coefficients before and after the adjustment for energy, are depicted in Table 3. Clinical outcome results reported below are those obtained after PSM, unless noted otherwise.

**Table 3:** Clinical outcomes of 61,437 internal medicine inpatients aged 18 years or older and fasting for more than 10 days in Japan, January 2011 to September 2020

	Before PSM		After PSM			OR/regression coefficient^e^ (95%CI)	
	Non-ILE group (*n* = 41,819)	ILE group (*n* = 19,618)	Non-ILE group (*n* = 19,602)	ILE group (*n* = 19,602)	*p*-value	Unadjusted	Adjusted^f^
*Primary endpoint*							
In-hospital mortality	11,712 (28.0)	3976 (20.3)	5272 (26.9)	3972 (20.3)	< 0.001^c^	0.69 (0.66 to 0.72)	0.71 (0.68 to 0.75)
*Secondary endpoint*							
IV catheter infection	378 (0.9)	213 (1.1)	178 (0.9)	213 (1.1)	0.08^c^	1.20 (0.98 to 1.46)	1.05 (0.85 to 1.29)
Deteriorated ADL^a^	3054 (12.0)	1458 (10.8)	1541 (12.5)	1456 (10.8)	< 0.001^c^	0.85 (0.79 to 0.92)	0.88 (0.81 to 0.95)
LOS^b^, mean (SD), *days*	44.2 (39.1)	42.3 (34.0)	43.1 (36.1)	42.3 (34.0)	0.045^d^	− 0.8 (− 1.6 to 0.0)	− 1.8 (− 2.6 to − 1.0)
Readmission^b^	2227 (7.4)	1132 (7.2)	1073 (7.5)	1132 (7.2)	0.42^c^	0.96 (0.88 to 1.05)	0.95 (0.87 to 1.04)
Total medical cost, mean (SD), US$	21,084 (23,729)	21,019 (18,486)	21,402 (24,981)	21,009 (18,439)	0.08^d^	− 393 (− 822 to 48)	− 860 (− 1252 to − 47)

Data presented as numbers of patients (percentages), unless otherwise indicated

*Abbreviations*: *ADL*, activity of daily living; *CI*, confidence interval; *ILE*, lipid injectable emulsion (soybean oil-based); *IV*, intravenous; *LOS*, length of stay; *OR*, odds ratio; *PSM*, propensity score matching

^a^Denominator was patients discharged alive who had Barthel Index data at both admission and discharge (before PSM, 25,501 in the non-ILE group and 13,445 in the ILE group; after PSM, 12,311 in the non-ILE group and 13,434 in the ILE group)

^b^Denominator was patients discharged alive (before PSM, 30,107 in the non-ILE group and 15,642 in the ILE group; after PSM, 14,340 in the non-ILE group and 15,641 in the ILE group)

^c^Based on the chi-square test

^d^Based on the Student *t*-test

^e^Based on multiple regression analysis or multivariate logistic regression analysis

^f^Adjusted by adding the mean daily energy dose prescribed for days 4 to 10 to variables (day 1 regarded as the day fasting started)

### Primary endpoint

**Fig. 2:**
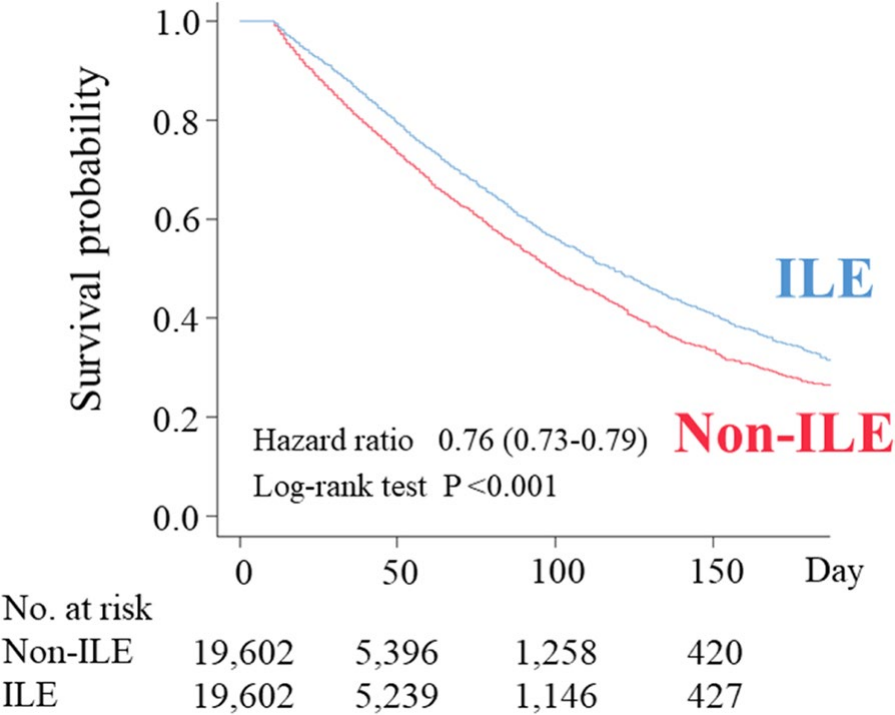
Kaplan-Meier survival curves for in-hospital mortality of internal medicine inpatients aged 18 years or older and fasting for more than 10 days in Japan, January 2011 to September 2020, after propensity score matching. The results expressed as the hazard ratio (95% confidence interval) of in-hospital mortality for the ILE group compared to the non-ILE group. Curves demonstrate a significantly lower hazard for in-hospital mortality for the ILE group vs. the non-ILE group (HR, 0.76; 95% CI, 0.73–0.79; *p* < 0.001). *Abbreviations*: ILE, lipid injectable emulsion (soybean oil-based)

The unadjusted odds of in-hospital mortality were significantly lower for the ILE group than for the non-ILE group (OR, 0.69; 95% CI, 0.66–0.72; *p* < 0.001). After adjusting for the energy variable, the OR for in-hospital mortality showed the same trend (OR, 0.71; 95% CI, 0.68–0.75). The Kaplan-Meier curves demonstrated a significantly lower hazard for in-hospital mortality for the ILE group vs. the non-ILE group (HR, 0.76; 95% CI, 0.73–0.79; *p* < 0.001) (Fig. 2).

### Secondary endpoints

There was no significant difference between the 2 groups in terms of intravenous catheter infections (1.1% in the ILE group vs. 0.9% in the non-ILE group; unadjusted OR, 1.20; 95% CI, 0.98–1.46; *p* = 0.08). The unadjusted odds of deteriorated ADL in the patients who were discharged alive were significantly lower for the ILE group than for the non-ILE group (OR, 0.85; 95% CI, 0.79–0.92; *p* < 0.001). The unadjusted regression coefficient for LOS in the ILE vs. the non-ILE group was − 0.8 (95% CI, − 1.6–0.0; *p* = 0.045), and the coefficient adjusted for the mean daily energy dose variable was − 1.80 (95% CI, − 2.6 to − 1.0).

The mean (SD) total medical costs were $21,009 ($18,439) in the ILE group and $21,402 ($24,981) in the non-ILE group (*p* = 0.08), with the ILE group vs. non-ILE group unadjusted regression coefficient of − $393 (95% CI, − $822–$48). However, after adjustment for the prescribed mean daily energy dose on days 4 to 10, the regression coefficient of total medical costs was − 860 (95% CI, − $1252 to − $47).

### Sensitivity analysis

The VIFs of patient characteristics and mean daily parenteral nutrition doses were all below 2.5, confirming that there was no multicollinearity between the variables (Additional file 1: Table S1). The ORs of in-hospital mortality and deteriorated ADL, after adjustment for patient characteristics (model 1), were 0.65 (95% CI, 0.62–0.68) and 0.77 (95% CI, 0.71–0.83), respectively (Additional file 1: Table S2). The regression coefficients for LOS were − 1.2 (95% CI, 2.0 to − 0.5) days in model 1 and − 2.1 (95% CI, − 2.8 to − 1.3) days after adding the adjustment for mean daily energy dose for days 4 to 10 in model 2, confirming the significant differences between the ILE group and non-ILE group in both models. As in the PSM analysis, there were no significant differences between the 2 groups in intravenous catheter infections and readmissions. Finally, the regression coefficients for total medical costs were − 411 (95% CI, − $776 to − $47) in model 1 and − $1244 (95% CI, − $1598 to − $850) in model 2, confirming that medical costs were significantly lower for the ILE group than for the non-ILE group.

## Discussion

To the best of our knowledge, this is the first large-scale cohort study investigating the impact of parenteral ILE use on clinical outcomes in a population of fasting internal medicine inpatients. In this study, we found that the patients in this population who were prescribed parenteral ILEs had significantly lower odds of in-hospital mortality and of deteriorated ADL than those who were not prescribed ILEs. In addition, the ILE group had a significantly shorter LOS than the non-ILE group. In contrast, the odds of having intravenous catheter infections did not differ significantly between the groups. Finally, after adjustment for the energy dose, the mean total medical costs for the ILE group were $860 lower than for the non-ILE group.

Lipids are one of 3 essential macronutrients, along with carbohydrates and proteins. The inclusion of ILEs in parenteral nutrition has been recommended in several guidelines [5, 18]. Despite this, this study confirmed that ILEs are not being used consistently as part of parenteral nutrition in Japan, as has been previously reported [7, 8]. In fact, we observed that twice as many patients received parenteral nutrition without ILEs as patients who received it with ILEs in Japan. There are several possible reasons why ILEs are often not part of the parenteral nutrition given in Japan. First, the only commercially available ILEs in Japan come in the form of soybean oil-derived products. However, these products may promote inflammatory reactions because they contain high levels of n-6 polyunsaturated fatty acids [19], and they may lead to a deterioration in immune function and an increased risk of infectious complications because of their inhibitory effect on phagocytosis [20]. Also, whereas 3-in-1 or total nutrient admixtures are used as the standard globally [21], no product containing all 3 macronutrients is commercially available in Japan. Given these product limitations, the complexity of adding ILEs separately to parenteral nutrition, and the possibility of insufficient training related to ILE use, clinicians in Japan may have been hindered in their prescribing of ILEs as part of parenteral nutrition.

We believe that the results of this study should encourage more clinicians in Japan to prescribe ILEs and thus promote the expansion of the market for ILEs. Moreover, significant gaps in ILE administration relative to published recommendations have been reported in other countries, including the USA [22]. On top of that, there has been a lack of studies investigating the prevalence and clinical impact of ILE use and involving real-world databases. Accordingly, further large-scale investigations should be performed to enhance the understanding of ILE use in large groups of patients and in real-world settings.

The results of this study have suggested that the use of ILEs during parenteral nutrition can have a positive impact on clinical outcomes, including reducing in-hospital mortality, deterioration of ADLs, and hospital LOS, and the results suggest this can be accomplished without increasing the risk of intravenous catheter infections. Taken together, these findings should raise awareness that many fasting hospital inpatients in Japan are not being prescribed ILEs during parenteral nutrition and that there are substantial benefits to adding ILEs to parenteral nutrition in hospital-based clinical practice. There are possible explanations for how the addition of ILEs to parenteral nutrition may have contributed to the positive clinical outcomes in our study. First, the essential fatty acids contained in ILEs are important components of the cell membrane and are known to play an important part in maintaining biological and physiological functions and as precursors of physiologically active substances [18]. Second, most patients who are fasting and require parenteral nutrition have impaired glucose tolerance, and ILEs can serve as effective alternative sources of energy as well as substances that exert protein-sparing effects [23, 24]. This protein-sparing effect of ILEs has been indirectly associated with improved clinical outcomes in fasting medical patients [25].

PSM was used in this study to mitigate the potential confounding by other variables when investigating the impact of the addition ILEs on clinical outcomes. The prescribed mean daily parenteral energy dose was not included as one of the covariates in the initial propensity score estimation, because it was anticipated that the total energy doses prescribed would be higher in the patients who received ILEs than in those who did not (simply based on the extra calories from the ILEs). Indeed, the ILE group did have a higher prescribed mean daily energy dose than the non-ILE group, and this was the case even after PSM was applied. Therefore, additional multivariate logistic regression and multiple regression analyses were performed, using the prescribed mean daily energy dose on days 4 to 10 as an additional explanatory variable, and then PSM was applied again to reassess the impact of the addition of ILEs on clinical outcomes. After this adjustment for the prescribed mean daily energy dose, the resulting ORs and regression coefficients for the clinical outcomes showed the same tendencies and statistical significance. Importantly, these results suggested the earlier findings and demonstrated that the addition of parenteral ILEs, independent of their contribution to higher energy doses, had significant beneficial effects on in-hospital mortality, deterioration of ADLs, and hospital LOS.

## Limitations

The present study has several limitations. First, this was a retrospective study. However, the sample size was large, and it can be challenging to perform a prospective, randomized controlled trial comparing outcomes in medical patients who received or did not receive ILEs. Second, despite efforts to control bias, unknown confounding factors or residual confounding might have been present. In the study, PSM was used to control for 17 potential confounding factors. However, residual confounding could have occurred, because data regarding disease severity and laboratory values could not be extracted from the database. Third, our findings were based on the information registered in a medical claims database. Because the database we used did not include information regarding the indications for parenteral nutrition for individual patients, we were unable to provide results concerning this characteristic in our patient population. Also, the database did not allow for a detailed cost analysis to investigate which factors enhanced cost-effectiveness. Moreover, the database had some missing data and may have contained entry errors. Finally, the use of ICD-10 codes to identify primary diseases is inferior to characterizing primary diseases prospectively. However, the use of CCI as a reliable and accurate measure of comorbidities has been validated in Japan [26]. Fourth, the ILEs prescribed to patients in this study were limited to soybean oil-based products because of the limited commercial availability of other products in Japan. Therefore, the effects of other ILEs, such as those derived from medium-chain fatty acids, olive oil, or fish oil, should be evaluated in future studies. Finally, the dose-dependent impact of ILEs on outcomes was not investigated as part of the study, because the mean daily doses of ILEs were too small. This issue should also be addressed in future studies.

## Conclusions

The results of this study should raise awareness that many fasting internal medicine inpatients in Japan are not being prescribed ILEs as part of parenteral nutrition. The addition of ILEs to parenteral nutrition in internal medicine inpatients not only improved clinical outcomes but also led to enhanced cost-effectiveness. Verification of these findings in observational or prospective studies would be necessary to confirm a direct causal relationship between the use of ILEs and positive clinical outcomes.

## Supplementary Information

**Additional file 1:** **Table S1.** Variance Inflation Factors (VIF) of patient characteristics and mean daily parenteral nutrition doses, for 61,437 internal medicine inpatients ages 18 years or older and fasting for more than 10 days in Japan, January 2011 to September 2020. **Table S2.** Sensitivity analysis of clinical outcomes of 61,437 internal medicine inpatients ages 18 years or older and fasting for more than 10 days in Japan, January 2011 to September 2020.

## Data Availability

The datasets used in this study were purchased from Medical Data Vision Co., Ltd. (MDV). The authors cannot share the data with any third parties or make the data publicly available due to confidential protection over the sharing of personal data.

## Abbreviations

ADL: Activities of daily living
BI: Barthel Index
BMI: Body mass index
CCI: Charlson Comorbidity Index
CI: Confidence interval
HR: Hazard ratio
ILE: Lipid injectable emulsion
JCS: Japan Coma Scale
LOS: Length of stay
OR: Odds ratio
PSM: Propensity score matching
SD: Standard deviations
VIF: Variance inflation factor
