# Supplementary material for: Clinical impact of lipid injectable emulsion in internal medicine inpatients exclusively receiving parenteral nutrition: a propensity score matching analysis from a Japanese medical claims database
Source: BMC Med. 2022 Oct 27;20:371. doi: 10.1186/s12916-022-02568-x (PMC9608912; doi:10.1186/s12916-022-02568-x)
Supplement: Supplementary file 1 — Additional file 1: Table S1. Variance Inflation Factors (VIF) of patient characteristics and mean daily parenteral nutrition doses, for 61,437 internal medicine inpatients ages 18 years or older and fasting for more than 10 days in Japan, January 2011 to September 2020. Table S2. Sensitivity analysis of clinical outcomes of 61,437 internal medicine inpatients ages 18 years or older and fasting for more than 10 days in Japan, January 2011 to September 2020. [file 12916_2022_2568_MOESM1_ESM.docx]

**Additional file 1: Table S1**. Variance Inflation Factors (VIF)^1^ of patient characteristics and mean daily parenteral nutrition doses^2^, for 61,437 medical inpatients ages 18 years or older and fasting for more than 10 days in Japan, January 2011 to September 2020.

| Variables | VIF |
| --- | --- |
| Age | 1.36 |
| Sex | 1.03 |
| Body mass index | 1.04 |
| Number of hospital beds | 1.05 |
| Year of admission | 1.02 |
| Type of admission | 1.11 |
| Primary disease | 1.87 |
| Charlson Comorbidity Index | 1.76 |
| Barthel Index | 1.55 |
| Japan Coma Scale | 1.29 |
| Albumin infusion^3^ | 1.10 |
| Blood transfusion^3^ | 1.09 |
| Respirator use^3^ | 1.08 |
| Dialysis^3^ | 1.04 |
| Nutrition support^3^ | 1.03 |
| Rehabilitation^3^ | 1.21 |
| Mean daily dose amino acid^2^ | 1.23 |
| Mean daily dose energy^2^ | 1.27 |

^1^Based on multiple regression analysis or multivariate logistic regression analysis; ^2^Between Days 4 and 10, with Day 1 regarded as day fasting started; ^3^Day of admission to Day 10, with Day 1 regarded as day fasting started.

**Additional file 2: Table S2.** Sensitivity analysis of clinical outcomes of 61,437 medical inpatients ages 18 years or older and fasting for more than 10 days in Japan, January 2011 to September 2020.

| *Clinical outcomes* | Non-lipid group  (n = 41,819) | Lipid group  (n = 19,618) | OR/Regression coefficient (95%CI) | | |
| --- | --- | --- | --- | --- | --- |
|  |  |  | Unadjusted | Model 1^3^ | Model 2^4^ |
| *Primary endpoint* |  |  |  |  |  |
| In-hospital mortality | 11,712 (28.0) | 3,976 (20.3) | 0.65 (0.63–0.68) | 0.65 (0.62–0.68) | 0.65 (0.62–0.68) |
| *Secondary endpoint* |  |  |  |  |  |
| IV catheter infection | 378 (0.9) | 213 (1.1) | 1.20 (1.02–1.42) | 1.18 (0.99–1.40) | 0.99 (0.83–1.19) |
| Deteriorated ADL^1^ | 3,054 (12.0) | 1,458 (10.8) | 0.89 (0.84–0.96) | 0.77 (0.71–0.83) | 0.77 (0.71–0.83) |
| Readmission^2^ | 2,227 (7.4) | 1,132 (7.2) | 0.98 (0.91–1.05) | 0.95 (0.88–1.02) | 0.92 (0.85–1.00) |
| LOS (days)^2^ | 44.2 (39.1) | 42.3 (34.0) | -1.9 (-2.6– -1.1) | -1.2 (-2.0– -0.5) | -2.1 (-2.8– -1.3) |
| Total medical cost (US $) | $21,084 (23,729) | $21,019 (18,486) | -$65 (-439–308) | -$411 (-776– -47) | -$1,244 (-1,598– -850) |

^1^Denominator was patients discharged alive who had Barthel Index data at both admission and discharge (25,501 in Non-lipid group and 13,445 in Lipid group); ^2^Denominator was patients discharged alive (30,107 in Non-lipid group and 15,642 in Lipid group; ^3^Model 1 adjusted for the 2 groups and patient characteristics (age, sex, body mass index, number of hospital beds, year of admission, type of admission, primary disease, Charlson Comorbidity Index, Barthel Index, Japan Coma Scale, and medical treatment day of admission through Day 10 [albumin infusion, blood transfusion, respirator use, dialysis, nutrition support, rehabilitation, and mean daily dose of amino acids prescribed on Days 4 to 10]), with Day 1 regarded as the day fasting started; ^4^Model 2 adjusted for all of the variables in Model 1 as well as mean daily energy dose prescribed on Days 4 to 10.

Data are presented as numbers (percentages) or means (standard deviation).

Abbreviations: ADL, activity of daily living; CI, confidence interval; IV, intravenous; LOS, length of stay; OR, odds ratio.
